# Do novel genes drive morphological novelty? An investigation of the nematosomes in the sea anemone *Nematostella vectensis*

**DOI:** 10.1186/s12862-016-0683-3

**Published:** 2016-05-23

**Authors:** Leslie S. Babonis, Mark Q. Martindale, Joseph F. Ryan

**Affiliations:** Whitney Laboratory for Marine Bioscience, University of Florida, 9505 Ocean Shore Blvd, St. Augustine, FL 32080 USA; Department of Biology, University of Florida, Gainesville, FL 32611 USA

**Keywords:** Novelty, Cnidocyte, Nematosome, Immune, Phagocytosis, RNA-Seq, *Nematostella vectensis*, Taxonomically-restricted genes

## Abstract

**Background:**

The evolution of novel genes is thought to be a critical component of morphological innovation but few studies have explicitly examined the contribution of novel genes to the evolution of novel tissues. Nematosomes, the free-floating cellular masses that circulate through the body cavity of the sea anemone *Nematostella vectensis*, are the defining apomorphy of the genus *Nematostella* and are a useful model for understanding the evolution of novel tissues. Although many hypotheses have been proposed, the function of nematosomes is unknown. To gain insight into their putative function and to test hypotheses about the role of lineage-specific genes in the evolution of novel structures, we have re-examined the cellular and molecular biology of nematosomes.

**Results:**

Using behavioral assays, we demonstrate that nematosomes are capable of immobilizing live brine shrimp (*Artemia salina*) by discharging their abundant cnidocytes. Additionally, the ability of nematosomes to engulf fluorescently labeled bacteria (*E. coli*) reveals the presence of phagocytes in this tissue. Using RNA-Seq, we show that the gene expression profile of nematosomes is distinct from that of the tentacles and the mesenteries (their tissue of origin) and, further, that nematosomes (a *Nematostella*-specific tissue) are enriched in *Nematostella*-specific genes.

**Conclusions:**

Despite the small number of cell types they contain, nematosomes are distinct among tissues, both functionally and molecularly. We provide the first evidence that nematosomes comprise part of the innate immune system in *N. vectensis*, and suggest that this tissue is potentially an important place to look for genes associated with pathogen stress. Finally, we demonstrate that *Nematostella*-specific genes comprise a significant proportion of the differentially expressed genes in all three of the tissues we examined and may play an important role in novel cell functions.

**Electronic supplementary material:**

The online version of this article (doi:10.1186/s12862-016-0683-3) contains supplementary material, which is available to authorized users.

## Background

Understanding the evolution of animal complexity requires an understanding of how new traits arise. Studies of novel trait evolution typically invoke either the origin of new genes [1, 2] or the evolution of new relationships among conserved genes [3] as the primary drivers of evolutionary innovation. Collectively, these studies suggest that novel genes can be generated in several ways, including: duplication and divergence of conserved genes, RNA-mediated duplication/retroposition, exon shuffling/mis-splicing of RNA and other modifications to coding sequence, horizontal transfer of genes from other taxa, *de novo* evolution of an open reading frame in previously non-coding sequence, and overprinting (i.e., transcription from multiple reading frames in the same gene) [1, 2, 4]. Indeed, this diversity of mechanisms underlying the generation of new coding sequence has led to the suggestion that novel genes are generated with high frequency across lineages [5]. In support of this, approximately 10-20 % of the genes in all sequenced genomes (as of 2010) comprise novel/taxonomically-restricted genes [6, 7], but the putative role of these abundant novel genes in driving evolutionary innovation is far from clear.

A recent study of drosophilids demonstrated that equally large proportions of novel (*Drosophila*-specific) and conserved genes (~30 % of each) were critical for viability, supporting the idea that recently evolved genes can be essential to the biology of the organism [8]. Several additional studies provide support for novel/taxonomically-restricted genes in generating lineage-specific morphological features (e.g., [9–13]) but surprisingly, most of these studies have identified only few novel genes to be contributing to the novel morphological trait of interest. Thus, despite their focus on the role of novel genes in the evolution of a novel trait, these studies actually reveal that the majority of the genes involved in evolutionary innovation are conserved. One challenge to studies of evolutionary novelty is the identification of a truly novel tissue to use as a model [14, 15]. Additionally, limited availability of data from closely related taxa means that many genes identified as “novel” may actually reflect sparse taxon sampling. Poor tissue sampling may pose an even bigger problem, leading to a general overestimation of the value of novel genes in the evolution of novel tissues merely because no other tissues were sampled. In fact, only few studies have explicitly examined the abundance and distribution of novel genes across cell/tissue types [12, 16, 17] and their results regarding the importance of novel genes have been conflicting.

Cnidarians have become a valuable model for the evolution of novelty because they possess an unequivocal example of a single-celled novelty: the cnidocyte (stinging cell). Cnidocytes are complex sensory/effector cells that exhibit significant morphological diversity across cnidarians (for a review of cnidocyte diversity, see [18]). Used primarily in prey capture and defense, cnidocytes are abundant in the tentacles, which are the primary feeding apparatus of cnidarians. Among anthozoans (e.g., corals and sea anemones), one of the notable synapomorphies is the presence of mesenteries [19, 20], multifunctional internal tissues of mixed developmental origin (endodermal and ectodermal) that contain gonadal and digestive cells as well as a diverse repertoire of other cell types, including cnidocytes. During feeding, the mesenteries become apposed to ingested prey items, presumably enabling the use of their cnidocytes and ectodermal gland cells to assist in immobilization/digestion of prey tissues [21]. The mesenteries of sea anemones in the superfamily Metridioidea [22] are modified to include threadlike distal extensions called “acontia” which are replete with cnidocytes. Interestingly, the suite of cnidocytes that populate the acontia can be distinct from that of the mesenteries, their tissue of origin [23]. While cnidarians may superficially appear simple, they clearly exhibit tissue-specific distributions of cell types resulting in a surprisingly high level of morphological complexity.

**Fig. 1:**
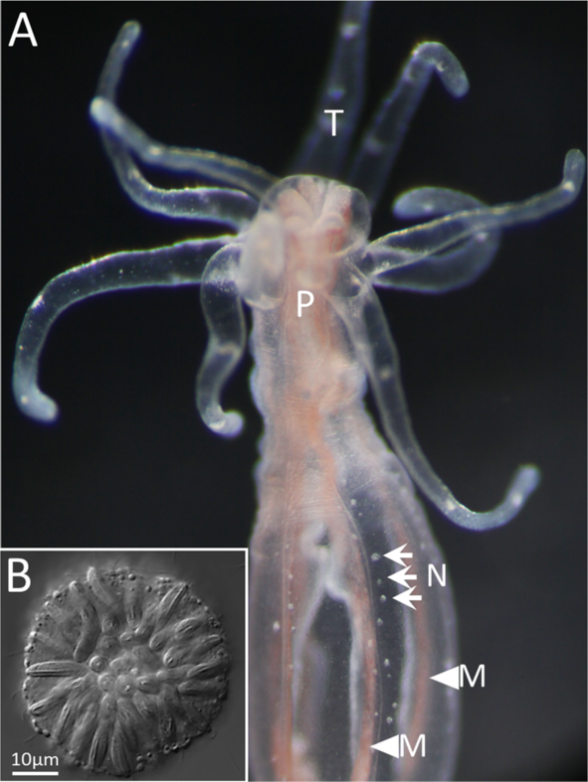
Nematosomes in *N. vectensis*. **a** A live image of a young adult polyp (10-tentacle stage); several nematosomes (N, arrows) are visible at rest along the internal surface of the body wall near the insertions of the mesenteries (M, arrowheads). The pharynx (P) and tentacles (T) are also visible. **b** A DIC optical section of an isolated nematosome

*Nematostella vectensis* (the starlet sea anemone) has emerged as an important model for diverse studies of cellular and molecular biology [24]; yet one of the most intriguing tissues in this animal remains largely unstudied: the nematosomes. First described by Stephenson in 1935 [25], nematosomes are an enigmatic free-floating tissue that circulates through the gastrovascular cavity (Additional file 1) in adult sea anemones, occasionally coming to rest along the internal surface of the body wall (Fig. 1a) [26, 27]. The presence of nematosomes in *N. vectensis*, *Milne-Edwardsia polaris* (later *N. polaris*), and *Milne-Edwardsia nathorstii* (later *N. nathorstii*), and their absence in other members of the Edwarsiidae led to the early designation of the nematosomes as the defining apomorphy of the genus *Nematostella* ([25, 28–30], as cited by [31, 32]). Despite this, little is known about the biology of this tissue. Nematosomes are small, multicellular masses of cells comprised largely of cnidocytes (Fig. 1b) and, like the acontia of metridoidean cnidarians, nematosomes are derivatives of the mesenteries [31]. Given the critical role of cnidocytes in the feeding behavior of sea anemones, early hypotheses of nematosome function suggested a role for this novel tissue in facilitating immobilization of ingested prey [27, 32]; yet support for this hypothesis has been inconclusive [33]. Histological studies of nematosomes confirmed that this tissue lacks gland cells leading to the conclusion that nematosomes play no role in digestion [33] but provided no convincing alternative function and no further information about the cell types present in these structures. The observation of abundant nematosomes in the jelly matrix surrounding the spawned egg masses [26, 34] led to a hypothesized role in protection of the developing embryos; yet, this hypothesis, too, has received mixed support [33, 35]. The long history of conflicting results regarding the putative function of nematosomes even led to the hypothesis that nematosomes play no role at all in the biology of *N. vectensis* [33], ultimately relegating the diagnostic morphological feature of the genus *Nematostella* merely to an intriguing curiosity.

Nematosomes are thought to develop continuously throughout the adult life of *N. vectensis* (albeit at a rate influenced by some undefined mechanism) making them a tractable model for understanding patterning and identity in an adult tissue. Since nematosomes are restricted only to the genus *Nematostella* (i.e., they have no phylogenetic homologs) and they are found only in adult polyps (i.e., they have no serial homologs), these unique structures are an unequivocal example of a phenotypic/tissue-level novelty [36, 37]. Whether this novel tissue expresses novel genes and cell types, however, is unknown. Herein, we (i) assess the function of this tissue by explicitly testing existing hypotheses from the literature, (ii) examine the morphology of this tissue for evidence of novel cell types, (iii) characterize the gene expression profile of the nematosomes (using RNA-Seq), and (iv) test hypotheses about the contribution of novel genes to novel structures by evaluating the distribution of novel and conserved genes in nematosomes and other tissues. Together, our results confirm that nematosomes comprise more than just a mass of sloughed cnidocytes and provide support for a novel role for this tissue in the innate immune system of *N. vectensis*. We further report that *Nematostella*-specific genes are indeed overrepresented in this *Nematostella*-specific tissue but these novel genes also comprise a large proportion of the upregulated transcripts across tissues, suggesting that novel genes may play important roles in the tissue-specific biology of all tissues.

## Results

### Nematosome development

**Fig. 2:**
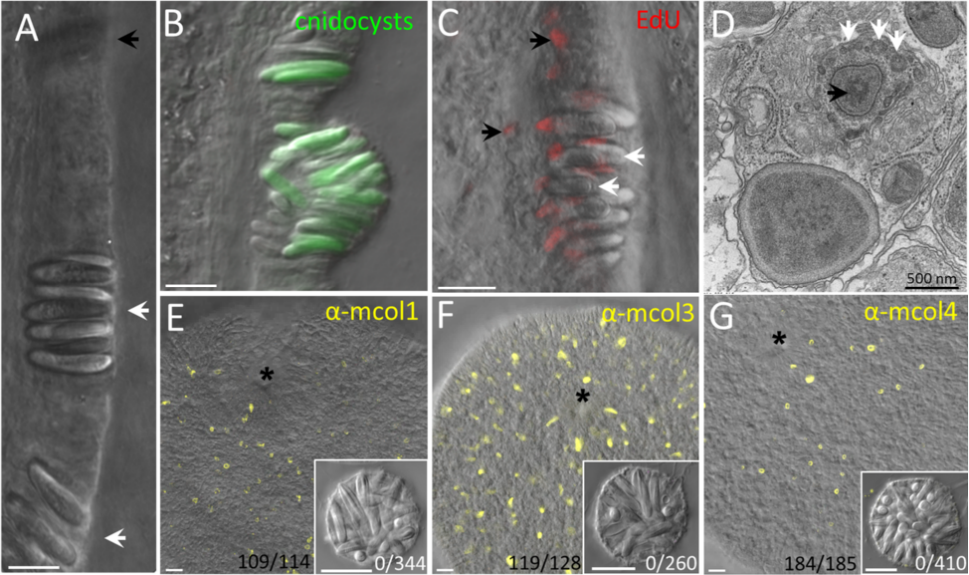
Nematosome budding. **a** Clusters of cnidocytes (white arrows) are visible in the ectodermal mesentery before budding (the black arrow indicates a cluster in a different focal plane). **b** A later stage in the budding process showing the abundance of mature cnidocytes (green; 143 μM DAPI) as the nematosome begins to protrude from the mesentery epithelium. **c** Proliferative nuclei (red, 100 μM EdU) are visible in the basal region of the epithelium, between and below the cnidocytes (white arrows). Proliferation also occurs outside of the budding zone (black arrows). **d** A developing cnidocyte from tentacle ectoderm; white arrows indicate the cnidocyst tubule which develops outside of and around the cnidocyst capsule (black arrow). **e**-**g** Immunohistochemistry performed in early planula stage embryos reveals abundant developing cnidocytes labeled with anti-mcol1, anti-mcol3, or anti-mcol4 antibodies (yellow). Nematosomes incubated at the same time in the same aliquot of each antibody lack staining (insets); the number of tissues (embryos or nematosomes) observed to have mcol^+^ cells is indicated. The blastopore of each embryo is indicated by * for orientation. All scale bars represent 10 μm unless otherwise specified

Nematosomes first appear in the body cavity of *N. vectensis* near the onset of reproductive maturity, after development of the ciliated tracts of the mesenteries (personal observations). Although material can be observed circulating through the body cavity of small polyps before maturation (Additional file 2A), this material is largely unicellular and does not contain cnidocytes. Similar material can be seen circulating through the cavity of adult polyps (Additional file 2B) but a comparison of the size of these particles with the nematosomes that also circulate in the cavity of adults makes clear that the former are not nematosomes. As noted by previous studies, nematosomes form by budding from the ectodermal (cnidocyte-rich) portion of the mesenteries [31]. Here, we demonstrate that nematosomes bud from regions of the mesenteries that are already rich in mature cnidocyte capsules (Fig. 2a, b) and the presence of cells in S-phase (labeled by EdU; Fig. 2c) suggests that the process of budding involves proliferation of the cells around the mature cnidocytes. Two pieces of evidence suggest that nematosome budding occurs only after completion of cnidocyte development: first, proliferative cells cannot be detected in this tissue. Despite the many thousands of nematosomes we assayed (*N* = 4 independent experiments on juvenile polyps or *N* = 3 independent experiments on egg masses, each representing thousands of nematosomes per experiment), we have observed only three nematosomes with proliferative cells (Additional file 3A). These results suggest new cells (cnidocytes or any other cell types) are not being made in the nematosomes after they leave the mesentery. Second, our attempts to identify developing cnidocytes directly have all failed. Using TEM, we have identified developing cnidocytes in the tentacle ectoderm from their unusual appearance (Fig. 2d) but have never seen developing cnidocytes in thin sections from nematosomes (e.g., Figs. 5 and 6). Also, using antibodies directed against three different minicollagens (mcol 1, 3, and 4), which are known to label only developing cnidocytes [38], we demonstrate that early planula stage embryos have abundant developing cnidocytes throughout the ectoderm while nematosomes assayed at the same time with the same antibodies, do not (Fig. 2e-g). In concert with the previous observation that nematosomes appear to lack cells expressing minicollagen RNA [39], these data suggest that nematosomes are shed from distinct regions of the mesenteries only after their cellular complement has been determined.

### Nematosomes subdue prey

**Fig. 3:**
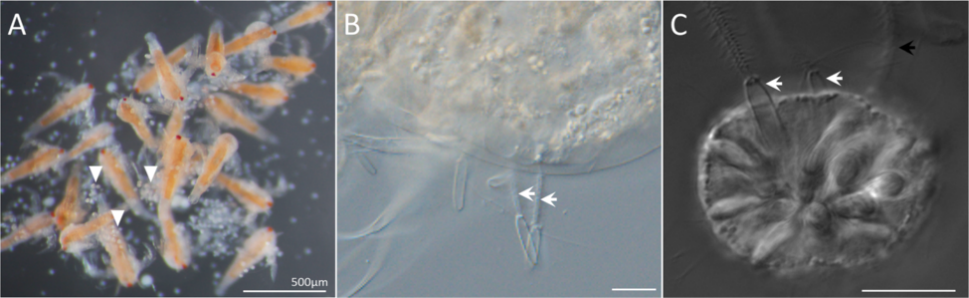
Live nematosomes are capable of subduing brine shrimp (*Artemia salina)*. (See also Additional file 4.) **a** Aggregates of nematosomes (arrowheads) are attached to subdued brine shrimp. **b** Higher magnification reveals the presence of two spent cnidocysts (arrows) protruding from the exoskeleton of the shrimp. **c** A high-magnification image of an isolated nematosome reveals two spent cnidocysts (white arrows). Black arrow indicates a spent cnidocyst in another focal plane. All scale bars represent 10 μm unless otherwise specified

To test the hypothesis that nematosomes are essentially non-functional waste material sloughed from the mesenteries [33], we assessed the capacity of nematosomes to subdue prey. To do this, we isolated live nematosomes from freshly spawned egg masses and concentrated them in a small amount of 1/3X filtered seawater (FSW) in a small glass dish. When recently hatched brine shrimp (*Artemia salina*) were introduced into the dish with the nematosomes, the shrimp became immobilized almost immediately (Additional file 4), at a rate consistent with the rate at which these animals are subdued by the tips of the tentacles in an adult *N. vectensis* polyp (Additional file 5). By contrast, 1/3X FSW alone failed to have any adverse effect on the shrimp (Additional file 6). High-magnification images taken at the end of the experiment reveal the presence of nematosome clusters adhering to the exoskeletons of the subdued shrimp (Fig. 3a) and at even higher magnification, discharged cnidocysts can be seen penetrating the exoskeleton (Fig. 3b). Likewise, a highly magnified image of an individual nematosome reveals the presence of multiple discharged cnidocysts (Fig. 3c). These data confirm that nematosomes possess the ability to subdue prey and that this is a result of cnidocyte firing.

### Nematosomes: more than just cnidocytes

**Fig. 4:**
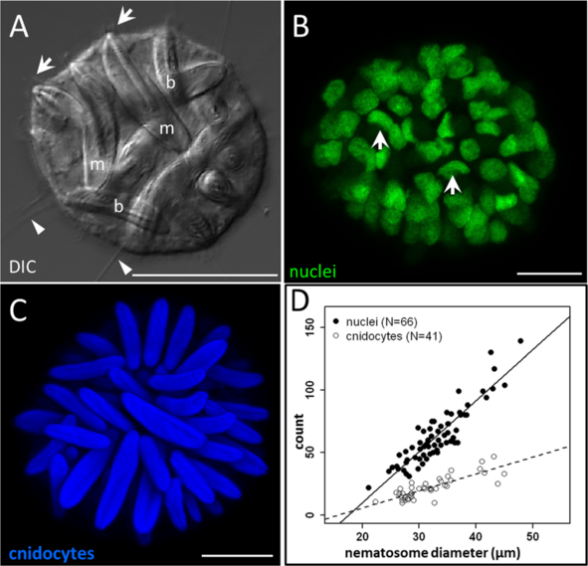
Nematosomes comprise multiple cell types. **a** A single DIC optical section through an isolated nematosome in which two types of cnidocyte are abundant: basitrichous isorhizas (b) characterized by a thin shaft extending nearly the full length of the capsule, and microbasic-p-mastigophores (m) which have a thick shaft that extends only half the length of the capsule and has a distinctive V-shaped notch [18]. Ciliary cones are visible (arrows) at the apex of two mastigophores and several long cilia (arrowheads) can be seen emerging from the perimeter of the tissue. **b** A 3D rendering of a confocal z-stack through a nematosome indicating nuclei (labeled with 1 μM DAPI; green). Nuclei in cnidocytes often appear semi-lunar in shape (arrows) as they conform to the shape of the cnidocyst capsule. **c** A 3D rendering of a confocal z-stack through another single nematosome showing the presence of abundant mature cnidocytes (labeled with 143 μM DAPI; blue). **d** The numbers of nuclei (an indicator of total cell number; closed circles) and cnidocytes (open circles) in individual nematosomes increase as a function of nematosome diameter but these relationships differ across the range of nematosomes studied (nuclei slope: 2.72, cnidocyte slope: 1.34; *p* < 0.001 ANCOVA). All scale bars represent 10 μm

Nematosomes have previously been reported to contain two of the three cnidocyte types found in *N. vectensis*: basitrichous haplonemes (“basitrichs”) and microbasic p-mastigophores [26]. We find both of these cells types in abundance across nematosomes (Fig. 4a). (Only a single spirocyte has been observed among the thousands of nematosomes we examined; Additional file 3B). To determine if other cell types (beyond mature cnidocytes) might be present in nematosomes, we counted nuclei (Fig. 4b) and cnidocyst capsules (Fig. 4c) in two independent samples of nematosomes, each spanning the range of nematosome size (*N* = 66 and *N* = 41 nematosomes, respectively). Both the total number of nuclei and the number of cnidocyst capsules increase linearly with size over the range of nematosomes examined (Fig. 4d; nuclei *R*^*2*^ = 0.82, cnidocytes *R*^*2*^ = 0.67, *p* < 0.001), but these relationships differ in slope (nuclei: 4.07, cnidocytes: 1.34). Because nematosomes do not contain developing cnidocytes (Fig. 2), these relationships suggest two things: first, all nematosomes have more nuclei than cnidocyst capsules and, therefore, must contain non-cnidocyte cells, and second, that large nematosomes contain more non-cnidocyte cells than do small nematosomes.

### Nematosome apical morphology is marked by abundant sensory cones

**Fig. 5:**
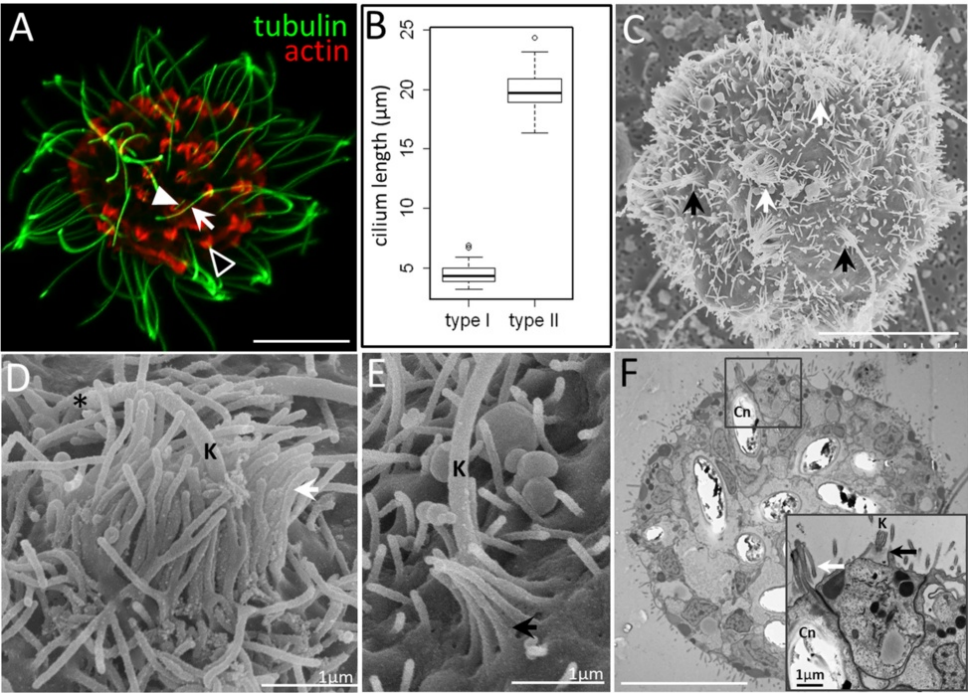
Ciliary structures on the surface of a nematosome. **a** Double labeling of F-actin (phalloidin; red) and acetylated-tubulin (green) indicates the presence of dense actin-rich apical cones (black arrowhead) and two types of cilia: type I, short (white arrowhead) and type II, long (white arrow). **b** Cilium length differs significantly between type I and type II cilia (ANOVA, *p* < < 0.001). **c** Numerous ciliary cones of different morphologies (arrows) are visible among the abundant microvilli covering the surface of a nematosome. **d** Type I ciliary cones (white arrows in A) are wide, assembled from multiple layers of stereocilia (white arrow), and surround a short central kinocilium (K; *indicates the distal tip of the cilium). **e** Type II ciliary cones are narrow and are composed of a single layer of stereocilia (black arrow) that surround a long kinocilium. **f** A thin section reveals the relationship of the ciliary cones and their underlying cell types. Higher magnification (inset) shows a type I ciliary cone (white arrow) at the apex of a cnidocyte (Cn) and a type II ciliary cone (black arrow) at the apex of a cell type with sub-apical vesicles of various size and electron density immediately adjacent to the cnidocyte. All scale bars represent 10 μm unless otherwise specified

In addition to cnidocytes, nematosomes are known to have abundant motile cilia [25, 26], although the nature of the cell type from which these cilia emerge has not been examined. Using fluorescence microscopy and an antibody directed against acetylated-tubulin, we confirm the presence of abundant cilia emerging from each isolated nematosome (Fig. 5a). Quantitative comparisons of cilium length (Fig. 5b) reveal the presence of two distinct types: Type I cilia are short, averaging 4.50 +/- 0.81 μm in length (Fig. 5a, white arrowhead) while type II cilia are nearly four times as long (19.84 +/- 1.71 μm in length; Fig. 5a, white arrow). Double-labeling with phalloidin (to detect F-actin) indicates the presence of numerous actin-rich apical cones (Fig. 5a, black arrowhead), previously described in tentacle epithelia of *N. vectensis* [40–42]. Using field-emission scanning electron microscopy (FE-SEM), we confirm the presence of at least two types of apical cones, associated with cilia of different length (Fig. 5c): type I cones have abundant short stereocilia organized into concentric whorls (Fig. 5d, white arrow) from which a short (type I) cilium emerges [43]. By contrast, type II cones are composed of a single ring of stereocilia connected by inter-ciliary links (Fig. 5e, black arrow) from which type II cilia emerge. Both types of ciliary cones were abundant in nematosomes of all sizes. Thin sections indicate that type I ciliary cones are found at the apex of cnidocytes while cells with type II ciliary cones are often found adjacent to cnidocytes (Fig. 5f) in a cell type that shares morphological features with the previously described cnidocyte support cells [44].

### Phagocytes: a new cell identified among nematosomes

**Fig. 6:**
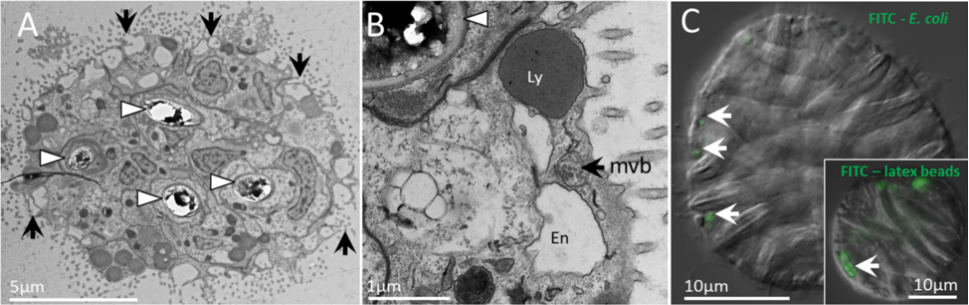
Phagocytes in nematosomes. **a** A thin section of a nematosome showing four cnidocytes (white arrowheads) and at least five cells with abundant apical vacuoles (black arrows). **b** At higher magnification, several organelles are visible: (mvb) multivesicular body, (Ly) lysosome-like vesicle, and (En) endosome-like vesicle. **c** An optical section through a single nematosome showing sub-surface localization of fluorescently-labeled *E. coli* (white arrows) and latex beads (inset)

Using TEM, we also identify an additional cell type in nematosomes with the morphology of a phagocyte. Thin sections reveal the abundance of large vacuolated cells in the periphery of an individual nematosome (Fig. 6a, black arrows). High-magnification imaging of one of these peripheral cells (Fig. 6b) reveals vesicles with the morphology of lysosomes, primary endosomes, and mature endosomes (multivesicular bodies), all within 1-2 μm of the apical membrane, consistent with the components of the endocytotic pathway in anthozoans [45]. Additionally, live nematosomes readily engulfed FITC-labeled heat-inactivated *E. coli* (Fig. 6c) and FITC-labeled latex beads (Fig. 6c, inset), confirming the capacity of nematosomes to phagocytose various types of particles. Together, these data provide the first evidence of phagocytotic capacity in nematosomes and suggest nematosomes may play a role in clearing the gastrovascular cavity of foreign particles.

### Constructing the nematosome transcriptome

One of the advantages of working with *N. vectensis* is the availability of genomic resources, including a publicly available reference genome [46] and several independent sources of transcriptome data (e.g., [47, 48]). However, tissue-specific transcriptome data are lacking and there have been no efforts to explicitly sequence the nematosomes. Considering the small size of the nematosomes and the fact that their abundance varies widely across individuals and throughout the year [26, 35], it is likely that this tissue was not well-represented in previous transcriptome studies. To gain insight into the molecular profile of nematosomes, we sequenced the complete set of transcripts expressed in the nematosomes and compared the expression profile of this tissue with that of the mesenteries (from which nematosomes arise) and the tentacles (another cnidocyte-rich tissue).

All three tissues (nematosomes, mesenteries, and tentacles) were collected from adult polyps and sequenced in triplicate using Illumina technology, resulting in over 100 M paired-end 100 bp reads per tissue. In order to identify novel (i.e., *Nematostella*-specific) transcripts with confidence, we chose to avoid the use of published gene models (since *ab initio* gene predictors may be biased toward identifying conserved sequences) and instead to assemble a reference transcriptome *de novo*, using reads from all three tissues and the *de novo* assembler Trinity [49, 50]. Erroneous assembly of numerous transcript “isoforms” is a common problem with *de novo* assemblers and a challenge to producing reliable differential expression results [51]; to avoid interpretation of these assembly errors as novel sequences, we aimed to minimize the number of assembled transcripts. To this end, we evaluated three reference transcriptomes assembled using different methods: a full transcriptome (TR333942) was assembled from the complete set of reads from all three replicates of all three tissues trimmed for adapters and low-quality bases using Trimmomatic [52], a second transcriptome (TR118377) was assembled from all the trimmed reads from single replicate of each tissue, and a third transcriptome (NvecRef32743) was assembled from a single replicate of each tissue after trimming and subjecting reads to error-correction using AllPaths-LG [53]. All raw reads and the three transcriptomes assembled from them have been deposited in the European Nucleotide Archive, Project Accession: PRJEB13676.

To evaluate the quality of our three transcriptomes, we first examined each for the number of transcripts and open reading frames (ORFs) they generated. To minimize the number of spurious ORF predictions, we used the default settings in Transdecoder (http://transdecoder.github.io/) which limits predictions to only those sequences with an ORF >100 amino acids (aa) in length. We further examined the number of expressed transcripts by mapping our complete set of reads back to each reference transcriptome using Bowtie2 [54]; aligned reads were then counted with the multiBamCov utility in BedTools [55]. Finally, we examined each transcriptome for completeness with CEGMA [56], which uses BLAST to search a set of 248 conserved eukaryotic genes against each assembled transcriptome. CEGMA scores represent the percent of the 248 conserved genes that were present in each assembly; assemblies with higher CEGMA scores are considered to be more complete.

The full transcriptome comprised 333,942 transcripts but only 9 % of these (29,690 transcripts) encoded ORFs > 100aa (Table 1), suggesting the majority of the assembled transcripts are very short. Additionally, each ORF-encoding transcript produced an average of 4.6 unique ORFs. While this resulted in 135,213 total predicted ORFs, the majority of these putative proteins (55 %, 74,503 transcripts) were estimated to have no expression (total count < 2 across tissues). Together, these data suggest that TR333942 does indeed contain a large number of erroneously assembled transcripts, however, this assembly also had the highest CEGMA score, as 242/248 (98 %) conserved eukaryotic genes from the CEGMA database were present in this transcriptome. Thus, TR333942 is useful for identifying complete transcript sequences but not for assaying differential expression across tissues.

**Table 1:** Comparison of transcriptome assembly statistics

	Trimmed only, *N* = 3 reps/tissue	Trimmed only, *N* = 1 rep/tissue	Trim + error correction, *N* = 1 rep/tissue
Transcriptome name	TR333942	TR118377	NvecRef32743
Assembled “transcripts”^a^	333,942	118,377	32,743
Transcripts with predicted ORFs^b^	29,690 (9 %)	19,461 (16 %)	17,346 (53 %)
Predicted ORFs^c^	135,213 (4.6X)	77,877 (4X)	27,511 (1.6X)
ORFs with no expression^d^	74,503 (55 %)	28,975 (37 %)	10,198 (37 %)
CEGMA score (complete) ^e^	242/248 (98 %)	241/248 (97 %)	207/248 (83 %)
CEGMA score (partial)	245/248 (99 %)	245/248 (99 %)	231/248 (93 %)

^a^Total number of contigs assembled using Trinity [49, 50]. ^b^Open reading frames (ORFs) ≥ 100 amino acids (aa) in length were predicted using Transdecoder (http://transdecoder.github.io). ^c^The average number of ORFs predicted from each transcript is listed in parentheses. ^d^Total number of transcripts with at least one ORF and abundance estimated at < 2 counts in at least one of the three sampled tissues; counts were assayed using Bedtools [55]. ^e^CEGMA scores are listed as % of the 248 conserved eukaryotic genes that form the CEGMA database [56] that were present in the indicated transcriptome (scores for complete and partial sequences are indicated)

The transcriptome assembled from one trimmed replicate of each tissue (TR118377) produced 118,377 transcripts, 16 % of which (19,461 transcripts) encoded long ORFs; this assembly, therefore, resulted in fewer erroneously assembled short transcripts than TR333942. However, those 19,461 transcripts produced 77,877 predicted ORFs, which equates to an average of approximately 4 unique ORFs per transcript. Like TR333942, TR118377 was characterized by a large number of sequences (28,975 transcripts) with no expression and also a very high CEGMA score, making this transcriptome also useful for identifying complete transcript sequences but not expression analyses.

Finally, the reference transcriptome assembled from trimmed/error corrected reads from a single replicate of each tissue (NvecRef32743) produced 32,743 transcripts, most of which (53 %, 17,346 transcripts) encoded long ORFs and produced only 1.6 ORFs per transcript. This resulted in 27,511 total predicted ORFs, 37 % of which (10,198 transcripts) were estimated to have no expression. The completeness of this reduced transcriptome was lower than either TR333942 or TR118377 (83 % vs 98/97 %) suggesting that error correction limited our ability to assemble full length transcripts. As an additional measure of assembly quality, we explicitly examined mapping concordance (percent of curated reads that mapped back to the NvecRef32743 assembly) following trimming and error correction (Table 2). Over 75 % of the reads from each sample mapped back to our reference assembly and error correction increased this number considerably (average 8.6 % increase in concordance). Given these metrics of assembly quality and our goal of confidently assembling truly novel transcripts we chose to use NvecRef32743 assembled from trimmed/error-corrected reads as our reference transcriptome for differential expression and orthology analyses. Our differential expression and qPCR analyses (see below) confirm that NvecRef32742 accurately represents the distribution of transcripts across tissues, supporting our decision to take this conservative approach.

**Table 2:** Error correction increases the mapping concordance for transcriptome NvecRef32743

Sample^a^	Raw reads (paired)	Reads remaining after trimming^b^	Reads remaining after trimming & Error Correction^c^	Reads aligned^d^ – Trimmed only	Reads aligned – Trimmed & Error Corrected
M3	49,390,203	49,377,414	46,888,223	39,768,569 (81 %)	43,249,696 (92 %)
M4	47,477,373	47,466,721	44,704,490	36,255,081 (76 %)	39,339,951 (88 %)
Mes	26,632,325	26,615,206	25,785,788	24,435,421 (92 %)	24,380,462 (95 %)
N1	47,788,007	47,770,601	44,558,275	36,611,388 (77 %)	39,656,864 (89 %)
N2	53,500,454	53,483,271	50,693,980	42,513,852 (79 %)	45,609,374 (90 %)
Nem	20,135,090	20,073,479	19,174,413	17,995,873 (90 %)	17,834,121 (93 %)
T3	47,683,996	47,670,541	44,585,027	27,963,673 (77 %)	39,854,556 (89 %)
T4	50,314,893	50,300,293	47,474,608	40,154,724 (80 %)	43,557,953 (92 %)
Ten	21,803,901	21,788,366	20,983,324	19,474,441 (89 %)	19,384,395 (92 %)

^a^Samples M3, M4, Mes are from mesenteries; N1, N2, Nem are from nematosomes; T3, T4, Ten are from tentacles. ^b^Trimming was performed using Trimmomatic [52]. ^c^Error correction was performed using the ErrorCorrectReads utility from AllPaths-LG [53]. ^d^Alignments were performed using Bowtie2 [54]

To ensure all assembled transcripts in our reference transcriptome were from *N. vectensis*, we used the program alien_index (https://github.com/josephryan/alien_index) to identify putative foreign/contaminating transcripts. From the assembled transcriptome of 32,743 transcripts, we identified 82 sequences of potential foreign origin and examined each manually using blastn in the published *N. vectensis* genome. Those sequences with alien index scores greater than 0, i.e., with a high likelihood of foreign origin (*N* = 82 sequences, available in Additional file 7) that also had high quality BLAST hits in the genome (>70 % identity and >200 bp in length, *N* = 45 sequences) were retained for further analysis; all other sequences were considered putative contaminants (*N* = 37 sequences) and were removed (see summary of alien index analysis in Additional file 8). In summary, our cleaned reference transcriptome consisted of 32,706 *N. vectensis* transcripts which encoded 17,313 predicted ORFs, 2150 of which lack models in the published JGI database (Table 3; Additional file 8).

**Table 3:** Reference transcriptome NvecRef32743 statistics

Total number of raw reads pooled for *de novo* assembly	137,142,632
Assembled “transcripts”	32,743
Transcripts remaining after alien index analysis	32,706
Transcripts encoding an ORF > 100aa in length	27,511
Transcripts with non-0 expression and ORF >100aa	17,313
New transcripts (not predicted from the genome)	2,150
Mean transcript length in bp (*N* = 32,706/17,313)	1,058/1,646
N50 in bp (*N* = 32,706/17,313)	1,919/2,282
GC content (*N* = 32,706/17,313)	44.56 %/45.5 %

Our goals for differential expression and orthology analyses were three-fold: first, we wanted to generate a molecular profile of the nematosomes by describing the types of genes expressed in this tissue with a specific focus on any novel transcripts that might have been missed in previous sequencing projects. Second, we wanted to explicitly test the hypothesis that the nematosome transcriptome comprises a subset of the genes expressed in the mesenteries (from which nematosomes arise) and further, that nematosomes share more genes in common with the mesenteries than with the tentacles. Third, we aimed to evaluate the hypothesis that *Nematostella*-specific genes are restricted to (or overrepresented in) this novel tissue.

### Nematosomes are molecularly distinct from mesenteries and tentacles

**Fig. 7:**
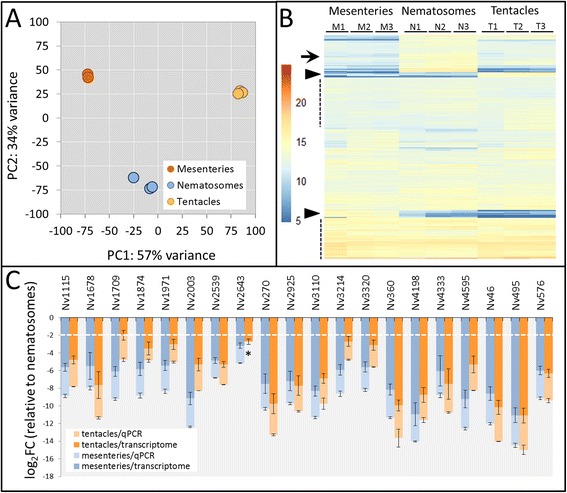
Expression analyses in three *N. vectensis* tissues. **a** Principal components plot indicating variation within and among tissue types. PC1 describes 57 % of the variation among tissues and PC2 describes 34 % in the top 1000 expressed transcripts. **b** Heatmap indicating gene expression (variance stabilized counts) of the top 1000 expressed transcripts. Transcripts are clustered using the Ward’s D method (see Additional file 9); warmer colors indicate higher expression. Dotted lines indicate clusters with ubiquitous expression across tissues, the arrow indicates a cluster with high expression in nematosomes only, and arrowheads indicate clusters with high expression in only mesenteries or tentacles. **c** Nineteen out of twenty transcripts identified by RNA-Seq as being significantly upregulated in nematosomes relative to both other tissues were also found to have significantly lower expression in the mesenteries and tentacles, relative to nematosomes, using qPCR. Expression of Nv2643 in the tentacles (indicated by *) is not significantly different from 0. Dotted lines indicate 2(log_2_)-fold change in expression between nematosomes and the other tissues. Mean ± SD

We used principal components analysis to assess variation among replicates and across tissues. While variation was highest among the nematosome samples, all three tissues formed distinct clusters along the first two components (Fig. 7a), confirming that variation among tissues exceeds variation among replicates and that the expression profiles of our three tissues are indeed distinct. To examine differences in gene expression across tissues, we performed hierarchical clustering based on fold-change expression differences in variance stabilized count data using DESeq2 [57] (See Additional file 9 for statistical methods). For this study, we consider only those transcripts with ≥ |2|(log_2_) fold-change differences between tissues with a false discovery rate of < 0.05 as differentially expressed. While numerous transcripts had largely ubiquitous expression across tissues, we also identified clusters of transcripts that were highly expressed exclusively in the nematosomes and clusters of transcripts with high expression in either mesenteries or tentacles (Fig. 7b).

To validate our differential expression patterns, we examined the expression of several transcripts found to be upregulated in the nematosomes using qPCR in independent samples (*N* = 3) of each tissue. We randomly selected twenty transcripts from the set of 528 transcripts identified as being upregulated in nematosomes for quantitative PCR analysis. Nineteen of these twenty transcripts exhibited significantly lower expression (≥2 log_2_ fold lower) in both the mesenteries and tentacles relative to the nematosomes (Fig. 7c). Together, these data suggest that our approach was effective at identifying differentially expressed transcripts across tissues.

### Lineage-specific genes are overrepresented in nematosomes (and all tissues)

**Fig. 8:**
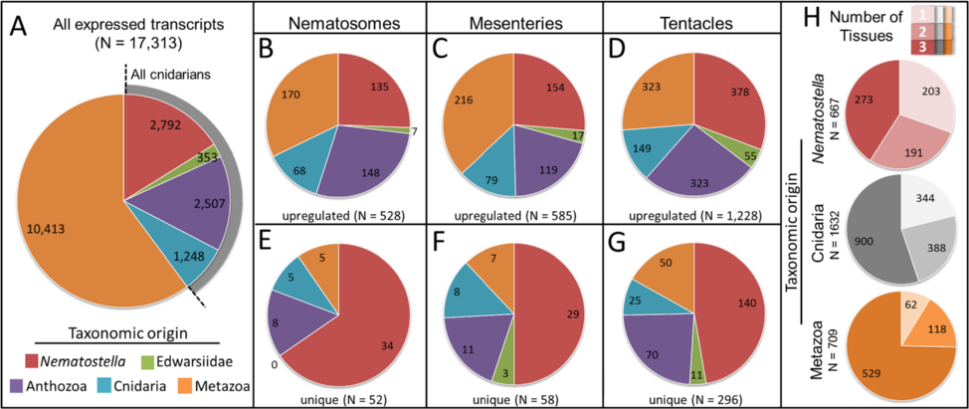
Analysis of taxon-restricted gene expression. **a** Of 17,313 expressed transcripts identified in this study, approximately 16 % (*N* = 2792) are *Nematostella*-specific, 2 % (353) are Edwardsiidae-specific, 14 % (*N* = 2507) are anthozoan-specific, and 7 % (*N* = 1248) are cnidarian-specific. The largest class of transcripts sequenced in this study (60 %) were found in at least one other group of metazoans (*N* = 10,413). As compared with the complete set of expressed transcripts, (**b**-**d**) differentially expressed transcripts were characterized by a higher proportion of *Nematostella*-specific sequences: 26 % in nematosomes and mesenteries (*N* = 135/528 and *N* = 154/585, respectively) and 31 % in tentacles (*N* = 378/1228). Shared/metazoan sequences comprised less than half of the differentially expressed transcripts in each of the three tissues. **e**-**g** *Nematostella*-specific sequences make up an even larger proportion of the uniquely expressed transcripts in each tissue: 65 % (*N* = 34/52) in the nematosomes, 50 % (*N* = 29/58) in the mesenteries, and 47 % (*N* = 140/296) in the tentacles. **h** Among the differentially expressed transcripts (illustrated in **b**-**d**), *Nematostella*-specific sequences were more likely to be expressed in only a single tissue (35 %, *N* = 203/667) than were pan-cnidarian sequences (illustrated in grey in panel **a**; 21 %, *N* = 344/1632) or shared/metazoan sequences (9 %, *N* = 62/709)

To characterize the types of transcripts upregulated in each tissue, we performed orthology analysis [58, 59] and assayed the distribution of transcripts of different taxonomic origins in each tissue. Specifically, we examined the distribution of *Nematostella*-specific genes, genes found only in representatives of the Edwardsiidae, genes found only among anthozoans, those found only among cnidarians, and conserved genes shared across metazoans (see Methods for a description of the specific taxa included in each orthology group). In the complete dataset (17,313 ORF-encoding transcripts), the largest proportion of transcripts (60 %, 10,413 transcripts) grouped with orthologs from across Metazoa, whereas *Nematostella*-specific transcripts comprised only 16 % of the dataset (2792 transcripts) (Fig. 8a). A previous study estimated the number of *Nematostella*-specific genes to comprise approximately 11 % of the genome [7], suggesting our efforts to assemble a reference transcriptome *de novo* was an effective method for identifying additional predicted genes.

Conserved/metazoan transcripts represented a smaller proportion of the upregulated genes in all tissues (Fig. 8b-d) than in the whole dataset (Fig. 8a). Of the 528 transcripts found to be upregulated specifically in nematosomes (≥2 log_2_ fold higher expression in nematosomes relative to both other tissues), only 32 % (170 transcripts) grouped with orthologs from diverse metazoans. *Nematostella*-specific transcripts comprised 26 % of the upregulated genes from nematosomes (135 transcripts) and fully 68 % of the transcripts in the upregulated genes in this dataset were cnidarian-specific (Fig. 8b). This pattern was conserved across tissues, with *Nematostella*-specific genes comprising 26 % of the upregulated genes from the mesenteries (Fig. 8c) and 30 % of the tentacle dataset (Fig. 8d), and the majority of the transcripts in both mesenteries and tentacles (63 % and 74 %, respectively) also grouping only with other cnidarian sequences. We further examined the subset of transcripts that were expressed uniquely in each tissue (Fig. 8e-g) to test the hypothesis that *Nematostella*-specific genes are expressed uniquely in a *Nematostella*-specific tissue. Of the 528 upregulated transcripts in the nematosomes (Fig. 8b), 52 were found to be expressed uniquely in this tissue (Fig. 8e). The majority of these uniquely expressed genes (65 %, 34 transcripts) were indeed *Nematostella*-specific but, surprisingly, 90 % (47 transcripts) of them grouped only with other cnidarian sequences. Genes expressed uniquely in both mesenteries and tentacles showed a similar pattern (Fig. 8f, g): in the mesenteries 50 % were *Nematostella*-specific and 88 % cnidarian-specific, and in the tentacles 47 % were *Nematostella*-specific and 83 % were cnidarian-specific. Thus, *Nematostella*-specific transcripts comprise a larger proportion of the uniquely expressed transcripts in nematosomes (65 %) than in both other tissues (50 % in mesenteries, 47 % in tentacles), but the largest number of *Nematostella*-specific transcripts was expressed uniquely in the tentacles (140 transcripts). Broadly, cnidarian-specific sequences dominate the set of differentially expressed transcripts in all tissues.

Finally, we ignored tissue type and examined the proportion of transcripts from each orthology group expressed in one or more tissues (Fig. 8h). Thirty percent of the differentially expressed *Nematostella*-specific transcripts (203/667 transcripts) were expressed only in a single tissue. By contrast, only 21 % of the cumulative group of cnidarian-specific transcripts (344/1632 transcripts) were expressed uniquely, as were only 9 % (62/709) of the conserved/metazoan transcripts (Fig. 8h). Together, these patterns provide further evidence that lineage-specific transcripts tend to be tissue-restricted whereas conserved/metazoan transcripts tend to be expressed across multiple tissues.

### Functional annotation supports a role in immunity

To characterize the suite of differentially expressed genes from each tissue, we used gene ontology (GO) functional annotation implemented in R [60] (see Additional file 10 for full GO reports.). First, we examined the functional annotation of the ten transcripts with the highest expression in each tissue (Table 4). While nematosomes are characterized by transcripts with extracellular/immune function (e.g., golgi autoantigen, hemicentin, thrombospondin), the highest-expressed transcripts in mesenteries were almost exclusively digestive enzymes (e.g., trypsin, chitinase). Intriguingly, highly expressed transcripts from tentacles tended to be unannotated sequences although two sequences appear to be involved in vertebrate immunity: hemicentin and rhamnose binding lectin. These results are consistent with a role for the nematosomes in the immune biology of *N. vectensis* and also suggest, surprisingly, that the functional identity of the nematosomes may be more similar to that of the tentacles than to the mesenteries.

**Table 4:** Pfam/Conserved domain analysis of the top expressed transcripts from each tissue

Tissue	TrID	GrID	JGI ID	Pfam/CD(s) ^a^	Description
nematosomes	Nv270	M	200843	Myosin_tail_1, Cast, TPR_MLP1_2, Mplasa_alph_rch	golgi autoantigen B1
	Nv46	N	239786	cadherin	uromodulin
	Nv1832	C	N/A	Calponin homology (CH) domain, Spectrin repeat, SMC_prok_A, PRK02224	utrophin
	Nv495	C	246793	reprolysin, pep_M12B_propeptide, ADAM_spacer1 super family, TSP_1, FN3, ZnMc super family, ADAM_CR super family	ADAMTS
	Nv791	C	27059	ZnMc_adamalysin_II_like, reprolysin, pep_M12B_propeptide, F5_F8 type C, disintegrin, ADAM_CysRich, TSP_1, FReD super family, EB	hemicentin/thrombospondin
	Nv126	C	242847	N/A	uncharacterized
	Nv354	M	119733	Homeobox KN domain, PRK12323 Sox_C_TAD super family	Meis2 transcription factor
	Nv111	N	200034	N/A	uromodulin
	Nv1971	A	96874	FN3, TLD, MAM, F5_F8_typeC, Ion_trans_2	sidekick-2 (immunoglobulin)
	Nv1874	M	21107	PKD channel, REJ, PLAT_polycystin, PKD, GPS, F5_F8_typeC, WSC, DUF4271 super family	cation channel
mesenteries	Nv2	M	39271	CstA, PLA2c	Phospholipase A2
	Nv151	M	205229	PHA03307, DUF1943, VWD, Vitellogenin_N	vitellogenin precursor
	Nv169	M	180912	CBM_14	chitin binding protein
	Nv224	M	168629	GH18_chitolectin_chitotriosidase, Glyco_18, Retinal, PHA03307, DUF2237 super family	chitinase
	Nv274	M	109239	ND2 super family, Tryp_SPc, SR	chymotrypsin
	Nv174	M	105779	Atrophin-1, Tryp_SPc, ShKT	chymotrypsin
	Nv158	N	246653	LDLa, UPF0104 super family, Tymo_45kd_70kd	uncharacterized membrane protein
	Nv215	M	15302	ZnMc_MMP, PG_binding_1, ShKT, Peptidase_M10	matrix metalloprotease
	Nv9181	M	246069	Mito_carr, PTZ00169	ADP,ATP carrier protein
	Nv896	C	98917	ZnMc_astascin like, ShK toxin domain, astacin (Peptidase family M12A), prolyl 4-hydroxylase	matrix metalloprotease
tentacles	Nv302	C	N/A	DnaJ_zf, GPS, TIG, Glyco_hydro_17 super family, REJ	egg jelly receptor
	Nv520	M	120496	PTKc, FN3, SEA, Ig_2, Pkinase_Tyr, IGc2	FGF receptor c
	Nv146	M	90289	KBL_like, Preseq_ALAS super family, BioF	aminotransferase
	Nv886	M	80526	Ig, Ig super family, Ig2_FGFR_like, Ig super family, I-set, IG_like, IG, IGc2	Immunoglobulin like activity
	Nv94	C	114661	SOUL	heme-binding protein
	Nv139	N	122916	Gal_lectin, FYDLN_acid super family	rhamnose binding lectin
	Nv325	M	202708	YtcJ_like	aminohydrolase
	Nv219	M	243257	Gln-synt_C, Gln-synt_N, PLN02284	glutamine synthetase
	Nv326	N	208719	TSP1, WSC super family	hemicentin
	Nv162	N	202441	DUF2457	uncharacterized

^a^ Protein family (Pfam) domains were identified from the EMBL-EBI database (http://pfam.xfam.org/) and conserved domains (CD) were identified from NCBI’s conserved domain database (http://www.ncbi.nlm.nih.gov/Structure/cdd/wrpsb.cgi). *TrID* internal transcript ID from NvecRef32743 *de novo* assembly, *GrID* orthology group, *N Nematostella*-specific, *A* anthozoan-specific, *C* cnidarian-specific, *M* shared/metazoan, *JGI ID* protein ID from the JGI database (http://genome.jgi-psf.org/Nemve1/Nemve1.home.html)

**Fig. 9:**
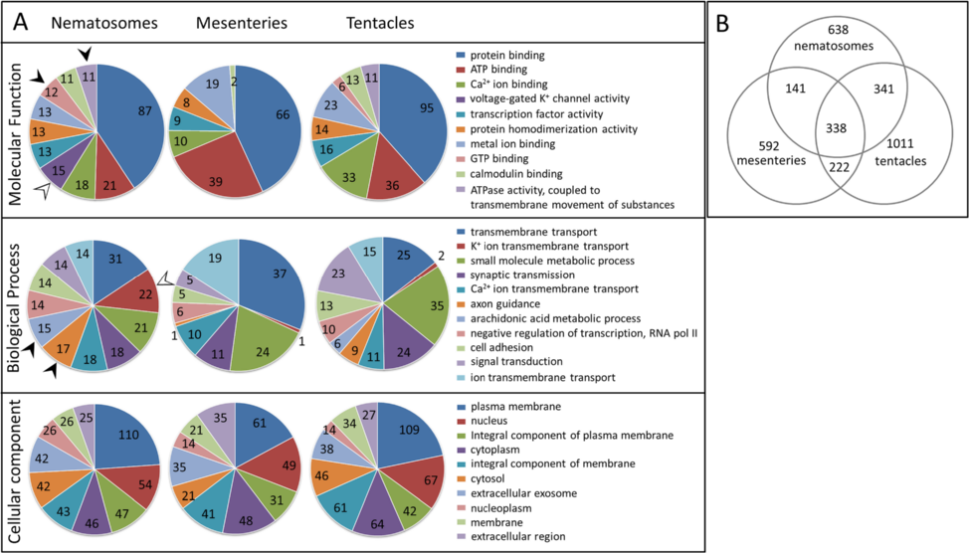
Gene ontology (GO) analyses of RNA-Seq data. **a** The top 10 GO terms from nematosomes for each GO category and the number of transcripts that map to them from other tissues. White arrowheads indicate GO terms unique (or nearly so) to nematosomes and black arrowheads indicate GO terms shared only by nematosomes and tentacles. **b** Venn diagram showing the number of GO terms shared among tissues

To further explore these hypotheses, we identified the top ten GO terms from nematosomes (for each GO category: molecular function, biological process, and cellular component) and compared the number of transcripts that mapped to each term across tissues (Fig. 9a). Two GO terms associated with potassium ion activity (MF: voltage-gated K^+^ channel activity, BP: K^+^ ion transmembrane transport) were found to be over-represented in nematosomes relative to both other tissues (Fig. 9a, white arrowheads), confirming the unique identity of this tissue and suggesting potentially novel cell physiology or cell signaling processes in this tissue. These analyses also revealed several GO terms that were common to nematosomes and tentacles but were absent from the mesenteries (Fig. 9a, black arrowheads), including terms associated with arachidonic acid metabolism, known for its important roles in cell signaling and inflammation [61, 62]. Overall, nematosomes were found to have 141 GO terms in common with mesenteries and 341 with tentacles (Fig. 9b, Additional file 10).

Finally, we assayed the set of upregulated transcripts (*N* = 528) from the nematosomes explicitly for genes associated with immunity using a reciprocal BLAST approach and a previously published database of stress genes from the *N. vectensis* genome [63]. This analysis revealed that over 20 % of the transcripts found to be upregulated in nematosomes (22 %, 116/528 transcripts) had reciprocal best BLAST hits with genes commonly associated with wound, pathogen, or chemical stressors (Table 5, Additional file 8). By comparison, only 17 % (101/585 transcripts) of the upregulated transcripts from the mesenteries had significant hits in this stress database and this value dropped to 13 % (161/1228 transcripts) for the tentacles. The stress categories “wound” and “pathogen” were associated with the most transcripts from each of the three tissues, but the distribution of transcripts annotated to these categories varied by tissue. Whereas transcripts associated with pathogen stress comprised the majority of the transcripts from nematosomes (75 %), less than 70 % of the transcripts from tentacles were associated with pathogen stress and only 56 % of the transcripts from mesenteries were associated with this stress category. Wound-associated transcripts comprised 33 % and 37 % of the transcripts from nematosomes and tentacles, respectively, and nearly 50 % of the transcripts from mesenteries. In summary, several pieces of data point to a novel immune capacity for the nematosomes.

**Table 5:** Abundance of candidate stress genes across tissues

	Nematosomes	Mesenteries	Tentacles
Transcripts assayed^a^	528	585	1228
Transcripts with reciprocal best hits in stress database^b^	116 (22 %)	101 (17 %)	161 (13 %)
Wound (*N* = 741)	38 (33 %)^c^	46 (46 %)	59 (37 %)
Pathogen (*N* = 1984)	87 (75 %)	57 (56 %)	109 (68 %)
Chemical (*N* = 187)	6 (2 %)	9 (9 %)	10 (6 %)

^a^The complete set of upregulated transcripts for each tissue. ^b^Database of putative stress-related transcripts identified by Reitzel et al [63] for the categories: Wound, Pathogen, and Chemical. For a list of genes in each tissue/class see Additional file 8. ^c^Values represent the number (percent) of the transcripts with reciprocal best hits in the stress database that were associated with the indicated stressor. The sum of these values exceeds the number of transcripts with reciprocal best hits because some transcripts had equally high BLAST hits in two different stress categories

## Discussion

### Evaluating the cell biology and function of nematosomes

Our data provide support for several previous hypotheses about the function of nematosomes, including their role in the prey capture and defense, and a novel potential role in the immune system of *N. vectensis*. In support of Hand [32], we confirm that the cnidocytes in nematosomes are capable of subduing prey (Fig. 3, Additional file 4), and that this function results from the firing of their abundant cnidocytes. However, like Williams [33], we find no evidence of gland cells in nematosomes; thus, our results are in conflict with the recent suggestion that the venom responsible for subduing prey derives from ectodermal gland cells adjacent to the cnidocytes in *N. vectensis*, rather than the cnidocytes themselves [64]. As noted in the early descriptions of nematosomes (summarized in: [32]), spirocytes are absent from this tissue, so it is possible, though speculative, that ectodermal gland cells work in tandem with spirocytes, which is why both are abundant in the tentacles.

Cnidocytes were once thought to behave as “independent effectors” responding autonomously to stimulus [65] but subsequent studies have revealed that this behavior may be modulated by chemical and mechanical stimuli [66] and by neuronal/synaptic input [67, 68]. The ultrastructural data presented in Fig. 5 suggest that nematosomes lack the sensory cell complexes necessary for mechanosensitization and neuronal modulation of cnidocyte discharge [69]; however, certain types of cnidocyte/support cell complexes are known to fire either as a result of physical contact alone or in response to combined physical and chemical stimuli [70]. Currently, we cannot discriminate between these two possibilities in nematosomes but future studies aimed at localizing chemoreceptors in the putative nematosome support cells will provide important insight on this topic. Furthermore, the absence of neurons from nematosomes may suggest that this tissue has evolved a means to limit the firing response of their cnidocytes to only physical stimuli, perhaps as a means to maximize firing success while nematosomes are “tumbling” around in the gastrovascular cavity.

Beyond the ability to subdue prey, we propose that nematosomes may also play a role in defense of the spawned egg masses. Unlike broadcast spawning species of sea anemone, *N. vectensis* produces egg masses bound by a jelly matrix [26, 34, 35]. Although the lifespan of nematosomes is reported by some to be < 21 h at room temperature [25], we routinely observed living nematosomes embedded in the egg jelly as planulae larvae begin to swim and emerge from the egg mass (>48 h after fertilization at 25 °C) (see Additional file 11 and [35]). Considering this long lifespan and the proximity to the developing embryos in the egg mass, nematosomes could reasonably play a role in defense of the spawned egg masses. Anecdotal observations of live killifish behavior also suggest that these potential predators may be deterred by cnidocyte discharge from the abundant nematosomes embedded in the jelly matrix (Adam Reitzel, pers. comm.). Together, these data suggest that nematosomes are an important component of the ecology of *N. vectensis*.

Our data also suggest that nematosomes may play an important part in the immune system of *N. vectensis*. Inflammation in cnidarians has been described as a global phagocytic response ([71], as translated by [72]). In support of this claim, the few previous studies of anthozoan immune function have demonstrated a dramatic increase in the number of mobile phagocytic cells following injury [72, 73]. Immune function has not been functionally characterized in *N. vectensis* but nematosomes might be an important reservoir for the phagocytes that comprise the putative inflammatory response in this species. Figure 6 demonstrates the propensity of cells in the nematosomes to engulf particles of various types (*E. coli* and latex beads), confirming the presence of phagocytes in this tissue. Furthermore, two of the top-expressed transcripts in the nematosomes share homology with uromodulin (Nv46 and Nv111), a glycoprotein known to be involved in protein-protein interactions associated with the inflammatory response of mammals [74, 75] and previously shown to be upregulated during wound-healing in *N. vectensis* [76]. Although the behavior of nematosomes during wound-healing/regeneration has not been studied, the presence of phagocytes suggests this tissue may play a role in clearing damaged tissue from a wound site. In this regard, it would be very interesting to determine if nematosomes aggregate at the site of a wound in *N. vectensis*.

We show that nematosome size is associated with its cellular composition (Fig. 4d); while cnidocytes make up half of the cells present in small nematosomes, they comprise only ~30 % of the cells in large nematosomes. We have also shown that nematosomes do not typically contain proliferative cells and likely do not undergo growth after they leave the mesentery epithelium, meaning that nematosome size is determined during the development and budding processes. Given that larger nematosomes have more non-cnidocyte cells than smaller nematosomes, and that at least some of these cells are phagocytes, we suggest that both nematosome size and budding rate may correlate positively with the animal’s perceived pathogen load. In this light, it would be interesting to raise *N. vectensis* polyps in the presence of antibiotics to determine if nematosome number decreases in this pathogen-free environment. We could further hypothesize that pathogen-related genes might be upregulated in nematosomes from polyps exposed to higher pathogen loads.

### Toward a molecular identity of nematosomes

The data presented herein represent the first tissue-specific transcriptome data from *N. vectensis*, an important model for cell biology and development, and the first molecular description of the nematosomes. Using these molecular resources, we evaluated two hypotheses about evolutionary innovation in *N. vectensis*. First, because nematosomes bud from the mesenteries and contain the same two types of cnidocytes found in the mesenteries (basitrichs and microbasic p-mastigophores), we hypothesized that the transcriptional profile of the nematosomes would constitute a subset of the transcripts expressed in the mesenteries. Differential expression, qPCR, and GO analyses independently verify the unique gene expression profile of the nematosomes (Figs. 7 and 8) and reinforce their putative role in immunity (Fig. 9, Tables 4 and 5) but also suggest that nematosomes may have more cell types or cell functions in common with tentacles than with mesenteries. These similarities in gene expression between nematosomes and tentacles may reflect the relative abundance of cnidocytes in these tissues, suggesting this dataset will be important for identifying novel markers of cnidocyte and support cell identity. Additionally, because mesenteries give rise to new nematosomes, the genes responsible for specifying this novel tissue at the onset of its development must be among the mesentery-specific dataset. Future studies aimed at identifying these nematosome developmental genes would be extremely valuable in understanding the evolutionary and developmental origin of this novel tissue. Thus, using comparative transcriptomics, we reject the hypothesis that nematosomes are merely sloughed portions of the mesenteries and suggest that nematosomes constitute a novel tissue with a unique identity and have characteristics consistent with cell functions that have not yet been characterized in *N. vectensis*.

The second hypothesis that we evaluated with these comparative transcriptome data was motivated by the assertion that lineage-specific genes are expected to be expressed in higher frequency in lineage-specific tissues [5, 77]. Given that nematosomes are unique to the genus *Nematostella*, we expected *Nematostella*-specific genes to be overrepresented in the nematosomes, relative to the two other tissues, one of which is specific to anthozoans (mesenteries) and the other is specific to cnidarians (tentacles). We found that *Nematostella*-specific genes comprised a larger proportion of the transcripts expressed uniquely in nematosomes than in either mesenteries or tentacles, but the largest number of *Nematostella*-specific transcripts was expressed uniquely in the tentacles. These results can be interpreted in several ways: first, the broader expression of conserved genes may simply reflect that a large proportion of the conserved genes we sequenced play a role in conserved functions that are common to all cells (i.e., “housekeeping” functions). Second, this pattern could reflect that conserved genes are expressed in cell types common to all three tissues, yet this hypothesis is harder to support considering that the only cell types known to populate all three tissues are cnidocytes. A third possibility is that this pattern reflects a greater level of pleiotropy in older genes. Indeed, novel genes are thought to arise rapidly [5] and have been shown by us and others to be uniquely expressed in adult tissues (e.g., [12, 16, 77]); whether these novel genes all acquire functions important for the phenotype of the tissue, however, is an unanswered question. Thus, despite the abundance of novel sequences produced, the percent of novel genes that actually take on a critical role in the biology of any given tissue may be relatively modest.

Although uniquely expressed transcripts were dominated by *Nematostella*-specific genes in all tissues, conserved/metazoan genes were among the top expressed transcripts from all three tissues (3/10 in nematosomes, 8/10 in mesenteries, and 5/10 in tentacles; Table 4). These data are interesting in the context of two recent studies of the role of novel genes in the evolution of novel hymenopteran social behaviors [12, 77]. Like us, Jasper et al [12] found that the proportion of lineage-specific genes in honey bees varies only little across tissue types but they suggest that the level of expression of these novel genes is responsible for driving the novel tissue identity. They show that the greatest proportion of gene expression in the venom/stinger and hypopharyngeal glands (both tissues with novel functions in the honey bee) is attributable to transcription of novel genes, but the patterns they describe appear to be driven by the exorbitantly high expression of a single gene in each tissue. In our study, the nematosomes (the novel tissue) were characterized both by a larger proportion of *Nematostella*-specific (novel) genes and a larger proportion of the top expressed genes were also found to be lineage-specific (pan-cnidarian).

### Are novel genes important?

It is well-established that both changes in gene regulation [3, 78] and changes in the coding sequences of conserved genes [4] drive morphological innovation but this does not preclude novel genes from also playing a critical role in this process [5]. In fact, there are several examples of novel proteins that are essential for defining the identity of the adult cell or tissue types (e.g., venom components [12], antimicrobial peptides [79], and structural proteins like minicollagen [80]). Interestingly, these novel products are largely secreted proteins that appear to have been incorporated into the terminus of an existing gene regulatory network. From an evolutionary perspective, the simplest way to link a novel protein into an existing regulatory network involves the smallest number of connections between the novel gene and the other components of the network [81] and this network connectivity issue may explain why duplicated genes at the ends of pathways may be maintained at a higher rate than duplicated transcription factors [13, 37]. In the context of this study, *Nematostella*-specific transcripts expressed uniquely in the nematosomes that encode secreted or structural peptides may well have a critical role in dictating the function or phenotype of this novel tissue.

## Conclusions

Cnidarian genome projects have revealed surprising levels of gene conservation between cnidarians and bilaterians; despite this, cnidarians have several cell and tissue types that are not found in other taxa. This study confirms that a novel tissue (nematosomes) plays an important role in the biology of *N. vectensis* and reveals, for the first time, that this novel tissue has a unique gene expression profile that differentiates it from its tissue of origin. Considering there are only 3-4 cell types present in this tissue, this unique molecular profile of nematosomes must be associated with the identity of only few cell types. Nematosomes, therefore, provide a valuable opportunity to evaluate the relationship between the expression of novel genes and the evolution of novel cell and tissue functions. Rather than being intriguing curiosities, novelties provide a unique opportunity to evaluate the mechanisms of evolution that result in the origin of new structures and have ultimately contributed to the morphological and genomic diversity exhibited in the animal kingdom.

## Methods

### Cnidocyte discharge assay

For discharge assays, live nematosomes were isolated from recently spawned egg masses using a modified protocol for isolating eggs from the egg jelly [82]. Egg jelly was dissolved by gentle shaking in 4 % cysteine (in 1/3X FSW) for 20 min at 25 °C in a 15 ml conical vial. Eggs were allowed to settle to the bottom of the conical vial and the supernatant (containing the nematosomes) was removed immediately and filtered first through a 70 μm nylon mesh strainer (BD Falcon 352350) to remove debris and then through a 10 μm nylon mesh filter (Millipore NY1002500) to collect nematosomes. Isolated nematosomes were washed in 3-5 changes of 1/3X FSW before use. To determine if the cnidocytes in nematosomes are capable of firing, live nematosomes were isolated and then concentrated in a small glass dish. Recently hatched brine shrimp (*A. salina)* were then introduced into this dish and their behavior was digitally recorded with a Canon digital camera mounted on Zeiss Discovery dissecting microscope. Higher magnification images were produced using either a Zeiss M2 compound microscope or a Zeiss LSM 710 confocal microscope.

### Tissue labeling and cell counts

For whole mount tissue analyses (polyps) and analysis of nematosomes isolated from spawned egg masses, tissues were immobilized in 7.14 % MgCl_2_ for 15 min at 25 °C, fixed briefly (~1.5 min) at 25 °C in 4 % paraformaldehyde with 0.2 % gluteraldehyde in phosphate buffered saline with 0.1 % Tween-20 (PTw) and then fixed for 1 h at 4 °C in 4 % paraformaldehyde in PTw. Fixative was removed from tissue with three washes in PTw and tissues were stored at 4 °C for up to five days before processing. To determine the total number of cells present in nematosomes of various size, we labeled nuclei with a 30-min incubation in 1 μM DAPI at 25 °C. Tissues were then washed in PTw and mounted in 80 % glycerol (in PBS) on Rain-X®-coated glass slides for imaging. To identify mature cnidocytes, we added a chelating agent (10 mM EDTA) to the fixative described above and labeled mature cnidocytes by incubation in 143 μM DAPI (in PTw) for 30 min at 25 °C, following the protocol of Szczepaneck et al [83]. For cell counts, confocal z-stacks were rendered into 3D images and digitally counted using Imaris software (Bitplane, Switzerland). Nuclei and cnidocytes were counted separately in different samples of nematosomes and diameter was measured digitally in each nematosome using the Zeiss LSM Image Browser software. To determine if the relationship between total cell number (indicated by nuclear counts) and nematosome size differed from that of cnidocytes over the range of nematosomes investigated, we performed Analysis of covariance (ANCOVA) using the R statistical computing environment [84].

To assess cell proliferation, tissues were incubated for 30 min at 25 °C in 100 μM EdU (in 1/3X FSW) and visualization was accomplished following the manufacturer’s protocol (Invitrogen C10340). To visualize developing cnidocytes, fixed/washed nematosomes and early planula stage embryos (72 h post fertilization at 16 °C) were incubated overnight at 4 °C with anti-minicollagen primary antibodies [38] diluted in PBS with 0.1 % TritonX (PTx) and 0.1 % bovine serum albumin to the following concentrations: mcol1 (1/300), mcol3 (1/500), mcol4 (1/1000). Primary antibody was removed with 3-5 washes in PTx and tissues were then incubated for one hour at 25 °C in goat-anti-guinea pig (mcol1 and mcol3) or goat-anti-rabbit (mcol4) secondary antibodies (Invitrogen A21450, A21245) at 1/250 in PTx. Unbound secondary antibody was removed with 3 washes (15 mins each) in PTx and tissues were mounted in glycerol. Similar methods were used to visualize the tubulin component of the cilia in nematosomes. Nematosomes were incubated overnight in anti-acetylated tubulin primary antibody (Sigma T6743) diluted 1/500 in PTx and with a goat-anti-mouse secondary antibody (Invitrogen A11001) diluted 1/250 in PTx. These tissues were counter-labeled with a 30-min incubation at 25 °C in a combination of 1 μM DAPI (in PTw) and phalloidin (Invitrogen A12380) diluted 1/200 in PTx to visualize nuclei and F-actin, respectively. Ciliary length in antibody-stained nematosomes was measured digitally using the Zeiss LSM Image Browser software and lengths were compared statistically using analysis of variance (ANOVA) in R [84]. To investigate the phagocytotic potential of the nematosomes, live isolated nematosomes were incubated overnight (~18 h) at room temperature with fluorescently labeled latex beads (L4530, Sigma Aldrich; final concentration of approximately 1/100 in 1/3X FSW) or FITC-labeled *E. coli* (Invitrogen E-2861; 2 mg/ml in 1/3X FSW). Excess particles were removed with three washes (15 mins each) in 1/3X FSW before tissues were fixed (as above) and cleared for imaging. All fluorescence imaging was performed on a Zeiss LSM 710 confocal microscope.

### Electron microscopy

For scanning electron microscopy (SEM), live nematosomes were removed from adult polyps and concentrated in a minimal amount (~300 μl) of 1/3X FSW which was pipetted directly onto a pre-wetted 0.2 μm GTTP filter (Millipore GTTP01300) mounted in a Swinnex filter holder in preparation for fixation. For TEM, whole polyps or spawned egg masses (containing nematosomes) were immobilized in 7.14 % MgCl_2_ for 15 min at 25 °C and collected/fixed in 1.5 ml sterile microcentrifuge tubes. All other fixation, preparation, and imaging methods were performed as previously described [42].

### Transcriptome sequencing and analysis

For sequence analysis, live nematosomes were removed from approximately 150-200 adult *N. vectensis* by making a small incision in the body wall of the polyp (near the pharynx) and then squeezing the nematosomes out into the surrounding 1/3X FSW in a small glass dish. Liberated nematosomes were then washed extensively in several changes of 1/3X FSW, immobilized in 7.14 % MgCl_2_ on ice for 15 min, and pooled into a single sample to recover enough material for one RNA-Seq replicate. This process was repeated three separate times (total ~500 adult polyps) to generate enough material for three independent replicates. For comparative transcriptomic assays, we also collected mesenteries (two mesenteries each from three adult polyps were pooled into a single sample; a total of three such samples were collected for sequencing) and tentacles (total of 30 tentacles from three adults, pooled; repeated for a total of three replicates). It should be noted that nematosomes develop from the mesenteries and although no budding nematosomes were observed at the time of collection of this tissue, we cannot exclude the possibility that our mesentery samples also contain nearly mature nematosomes (just prior to budding). All tissue samples (nematosomes, mesenteries, and tentacles) were transferred (with minimal 1/3X FSW) to sterile 1.5 ml microcentrifuge tubes before being snap-frozen on dry ice. Samples were stored at -80 °C for two weeks prior to processing. RNA was extracted and using a combination of Tri-Reagent (Sigma T9424; manufacturer’s protocol) and the RNAEasy Mini kit (Qiagen 74104; manufacturer’s protocol) and purified RNA samples were shipped to sequencing facilities on dry ice. cDNA library preparation and sequencing were performed commercially by SeqWright DNA Technology Services (Houston, TX, USA; 1 replicate of each tissue) or by the University of Florida’s Integrative Center for Biotechnology Research (2 replicates of each tissue).

Paired raw reads were trimmed for quality and to remove adapter sequences using Trimmomatic [52] and error-corrected using the ErrorCorrectReads.pl utility from AllPaths-LG [53]. (See Additional file 9 for bioinformatic commands.) Unpaired reads resulting from these processes were included with the left reads for subsequent assembly steps. Cleaned, error-corrected reads (paired and unpaired) were then assembled *de novo* using Trinity [49, 50]. To identify potential foreign/contaminating sequences, we performed an alien index analysis (https://github.com/josephryan/alien_index) following the methods of Gladyshev et al [85]. In brief, we downloaded whole proteome data from the UniProt website (http://www.uniprot.org/uniprot/; keyword 181 “proteome”) for diverse taxa: metazoans (SwissProt taxonomy ID: 33208; augmented by inclusion of the following pivotal taxa from NCBI unless otherwise indicated: *Amphimedon queenslandica*, *Hydra magnipapillata*, *Mnemiopsis leidyi*, *N. vectensis* from two sources: http://genome.jgi.doe.gov/Nemve1/Nemve1.home.html and www.cnidariangenomes.org, *Trichoplax adhaerens*), non-metazoan eukaryotes (ID: 2759), archaea (ID: 2157) and bacteria (ID: 2). We evaluated our reference transcriptome against this database using blastx and then calculated the alien index (AI) for all sequences as the ratio of the top non-metazoan blastx hit to the top metazoan blastx hit. All sequences for which AI > 0 (i.e., with better BLAST hits in non-metazoan taxa) were inspected individually to determine if they were part of the *N. vectensis* genome. Of these, only sequences with significant blastn hits in *N. vectensis* (>70 % sequence identity for ≥ 200 bp) were retained for further analysis.

Differential expression was assayed using DESeq2 version 1.11.23 implemented in R 3.2.3 following the recommendations in the User’s Guide [57]. DESeq2 uses a generalized linear model with negative binomial distribution and significance is assayed via the Wald test on normalized (to library size and sample variance) count data. Following significance testing, we excluded all data for which the false discovery rate (FDR/adjusted p-value) was ≥ 0.05 and fold change (log_2_FC) was < |2| in pairwise comparisons of any two tissues. Upregulated sequences were identified as those which were expressed significantly higher in the one tissue relative to both other tissues; uniquely expressed sequences were identified as the subset of upregulated sequences that were upregulated in a given tissue (FDR < 0.05, logFC > 2) and not expressed (average count across replicates within a tissue <2) in either other tissue. To confirm our differential expression analysis, we validated this approach using qPCR (below) on 20 randomly selected genes from the list of sequences upregulated in nematosomes. Upregulated sequences were functionally annotated against the NCBI *Homo sapiens* reference transcriptome (version 3/25/2015) using the AnnotationFuncs package in Bioconductor for R [60].

To identify novel transcripts from our dataset, we used a stringent approach to assigning sequence orthology based on reciprocal blastp searches of an expansive metazoan database using OrthoMCL [58, 59]. All 17,313 expressed transcripts with ORFs > 100aa were translated into all six reading frames (Transdecoder; http://transdecoder.github.io/) and all resulting predicted proteins were searched against a reference database containing two different sources of *N. vectensis* data: the JGI database (http://genome.jgi.doe.gov/Nemve1/Nemve1.home.html) and publicly available *de novo* transcriptome database from the Technau Lab at the University of Vienna (http://www.cnidariangenomes.org/). As there are currently no genomic data available for the two other species of *Nematostella* (*N. polaris* and *N. nathorstii*), our use of the term “*Nematostella*-specific” refers to transcripts present in a representative of the genus *Nematostella* (*N. vectensis*) and absent from a representative of the Edwarsiidae (*E. lineata*). This reference database also included transcriptome data from several other taxa (all available publicly, unless otherwise specified): anthozoan cnidarians - *Acropora digitifera*, *Aiptasia pallida* [86]*, Anthopleura elegantissima, Edwardsiella lineata, Fungia scutaria,* and *Nephthyigorgia* sp. [86]*;* other cnidarians *– Alatina alata* [86]*, Atolla vanhoeffeni* [86]*, Hydra magnipapillata, Podocoryna carnea* [86]*;* non-cnidarian taxa - *Amphimedon queenslandica* (Porifera)*, Branchiostoma floridae* (Chordata)*, Capitella teleta* (Annelida)*, Drosophila melanogaster* (Arthropoda)*, Homo sapiens* (Chordata)*, Mnemiopsis leidyi* (Ctenophora)*,* and *Trichoplax adhaerens* (Placozoa). Ortholog clustering was then assayed as follows: (i) *Nematostella*-specific sequences grouped only with other *N. vectensis* sequences from our reference transcriptome, the JGI database, and/ or the Technau database, (ii) Edwardsiidae-specific sequences grouped only with sequences from the *N. vectensis* databases and *E. lineata*, (iii) anthozoan-specific sequences grouped with sequences from *A. digitifera, A. elegantissima*, *A. pallida, F. scutaria* and/or *Nephthyigorgia* sp., (iv) cnidarian-specific sequences grouped with *A. alata, A. vanhoeffeni, H. magnipapillata,* and/or *P. carnea* sequences, and (v) conserved/metazoan sequences grouped with sequences from any of the following taxa: *A. queenslandica, B. floridanus, C. teleta, D. melanogaster, H. sapiens, M. leidyi,* or *T. adhaerens*. Predicted proteins that resulted from alternative ORFs in the same transcript sequence were subjected to orthology clustering independently but the final orthology of the transcript was assigned based on the highest value of the resulting categories (i.e., if any of the alternative proteins grouped with a conserved/metazoan sequence, the transcript was assigned to the conserved/metazoan group). (See Additional file 8 for a summary of the orthology and differential expression analyses.)

### Quantitative Real-Time PCR (qPCR) and standard PCR

Nematosomes were isolated from freshly spawned egg masses (as above), rinsed 3-5 times in 1/3X FSW and pooled into one sample in a sterile 1.5 ml centrifuge tube on ice; this was repeated three times. Three replicate samples consisting of all eight mesenteries from each of three adult polyps (nine polyps total) and three replicate samples of all sixteen tentacles from each of three adult polyps (same nine polyps used for mesentery collections) were also collected. All excised tissues were maintained on ice after dissection and processed within 1 h of removal from the polyp. Total RNA was extracted using Tri-Reagent and cDNA was synthesized from 1 μg of total RNA using the Advantage 2 RT for PCR kit (Clontech 639506), as previously described [87]. All primers were designed using the Primer3 utility in Geneious v.7.1.8 ([88] and qPCR primer efficiency was tested in a five-point standard curve (see Additional file 8 for primer sequences). qPCR was performed on 10 μl triplicates for each replicate sample using a Roche LightCycler 480 thermocycler and the manufacturer’s recommended SYBR mix (Roche 04887352001). Analysis of melt curves ensured our samples were free from genomic contaminants. Raw data were averaged (across biological replicates), normalized to the expression of housekeeping gene ATP synthetase, and are presented relative to the expression of each (normalized) transcript in nematosomes (set to 0). ANOVA was performed, as above, using R [84] and data are presented as means ± SD.

To further examine the specificity of the transcripts we identified as nematosome-specific with RNA-Seq and qPCR analyses, we attempted to amplify 10 nematosome-specific genes from three tissues not sampled for RNA-Seq - mixed-stage embryos (blastula through late planula), body wall, and pharynx – using standard PCR. Adult polyps were spawned and fertilized eggs were separated from egg jelly as above. Embryos were incubated at 16 °C for 48, 72, 96, 120, or 144 h to generate a sample of embryos from blastula, early planula, and late planula stages. Embryos were collected at the indicated stage, homogenized in Tri-Reagent by vigorous vortexing (30s at 25 °C) and stored at -80 °C until the remaining embryonic samples had been collected. Total RNA was extracted at the same time from all samples 24 h after the final collection of embryos and cDNA was synthesized (as above) immediately following RNA extraction. Body wall tissue was collected from adult polyps by bisecting animals at the aboral end of the pharynx and then freeing the body wall of the mesenteries as close to the point of insertion as possible. The entire aboral body wall was collected from three polyps and pooled into a single tube for RNA extraction and cDNA synthesis. Pharynx was collected from the same three individuals by removing the tentacles and hypostome at the oral end of the pharynx and freeing the pharynx from the surrounding body wall. Body wall and pharynx samples were stored on ice for no more than one hour after dissection and RNA extraction and cDNA synthesis were performed as described above. Ten sequences were randomly selected from the pool of nematosome-specific transcripts and primers were designed (as above) to amplify a 1000 bp region of the transcript. Standard PCR was performed using taq polymerase (New England Biolabs M0267L) following the manufacturer’s recommendations and amplification was performing for 35 cycles using an extension time of 1.5 min per cycle. PCR products were separated on a 1 % agarose gel and visualized using SYBRsafe gel stain (Invitrogen S-33102).

## Additional files

Additional file 1: Live nematosomes moving through the pharyngeal region of an adult polyp. (MP4 17442 kb) Additional file 2: (A) Small cellular material moving through the tentacle lumen of a primary/immature polyp. (B) Small cellular material and nematosomes moving through the tentacle of an adult polyp. (ZIP 51824 kb) Additional file 3: Further examination of nematosomes. (A) Proliferating cells in an individual nematosome labeled while it was circulating through the body cavity (red - proliferative nuclei, blue - non-proliferative nuclei, 1 μM DAPI). A total of three nematosomes have ever been observed to have proliferating cells via EdU. (B) (C-E) PCR confirms that nematosome-specific transcripts are uniquely expressed in nematosomes. Nine out of ten primers designed to amplify transcripts expressed uniquely in nematosomes failed to amplify products of the predicted size in cDNA samples extracted from mixed stage embryos, adult pharynx, and adult body wall. The product amplified using primers for Nv5804 (lane 1) appears to be ubiquitously expressed across all three tissues assayed. Actin was amplified as a positive control in lane 11. Faint bands in lanes 7-9 of the pharynx are of the wrong size and likely represent mis-priming. Lane 1: Nv5804, 2: Nv4198, 3: Nv13913, 4: Nv18469, 5: Nv5803, 6: Nv5749, 7: Nv9367, 8: Nv19938, 9: Nv18000, 10: Nv2134, 11: actin. See Additional file 8 for primer sequences. (PNG 1597 kb) Additional file 4: Live nematosomes can subdue recently hatched brine shrimp (*Artemia salina*). (MP4 49604 kb) Additional file 5: Cnidocytes in the tentacle tips of an adult polyp can subdue *A. salina*. (MP4 48606 kb) Additional file 6: 1/3X FSW alone (without nematosomes) has no effect on *A. salina*. (MP4 71560 kb) Additional file 7: Nucleotide sequences for 82 sequences with alien index > 0. (FA 51 kb) Additional file 8: Summary of everything. Sheet 1: Stress Gene Survey. List of tissue-specific targets and their reciprocal best blast hit (accession ID) from the dataset presented by Reitzel et al [63]. Included fields: Tissue, unique transcript ID (NvecRef ID), reciprocal best blastx hit (Hit), stressor type (pathogen, wound, or chemical). Transcripts with reciprocal best blast hits involved in both pathogen and wound stress are indicated as: Pathogen.Wound. Sheet 2: Transcriptome Summary. Summary of all 17,313 transcripts used for differential expression and orthology analyses. Each entry contains the following: unique transcriptome ID (NvecRef ID), transcript length (in nucleotides), orthology group ID (GrID): N – *Nematostella*-specific, E – Edwarsiidae-specific, A – anthozoan-specific, C – cnidarian-specific, M – conserved/metazoan, number of tissues (out of three) in which the transcript is expressed, differential expression result (UP/mes is upregulated in the mesenteries relative to both other tissues), mean counts across all tissues (AllMean) and across replicates within a tissue (MesMean, NemMean, TenMean), log2 fold change for all pairwise tissue comparisons (negative values indicate upregulation in the second tissue), adjusted p-values/false discovery rates for all pairwise comparisons, and raw counts for each tissue replicate. Sheet 3: Alien Index Results. Fields: unique transcriptome ID (NvecRef ID), alien index (AI), % identity and coverage of the top blast hit, and whether the sequence was deemed present in the genome (http://genome.jgi.doe.gov/Nemve1/Nemve1.home.html). Sheet 4: Primers. Sequences for all primers used in qPCR and standard PCR analyses. Primer sequences for each transcript are presented 5’ → 3’. (XLSX 4037 kb) Additional file 9: Commands for bioinformatic analyses and statistical methods. (TXT 5 kb) Additional file 10: Full GO annotation reports for upregulated transcripts by tissue. Sheet 1 (summary) includes full Blast2GO reports for all three tissues. Abbreviations: Mes – mesenteries, Nem – nematosomes, Ten – tentacles, Gene Symbol – NCBI approved gene symbol, GO ID – gene ontology identifier, BP (GO category) – Biological process, CC – cellular component, MF – molecular function, TrID – transcript ID (NvecRef transcriptome), HumRef – accession number (human orthologs) for NCBI’s Refseq database. (XLSX 183 kb) Additional file 11: Live nematosomes are still present (and moving) in the degenerating egg jelly after most of the *N. vectensis* planulae have emerged. (MP4 88967 kb)

## Abbreviations

1/3X FSW: 1/3X filtered seawater
ANCOVA: analysis of covariance
ANOVA: analysis of variance
BLAST: basic local alignment search tool
CEGMA: Core Eukaryotic Genes Mapping Approach
DAPI: 2-(4-amidinophenyl)-1H-indole-6-carboxamidine
DIC: differential interference contrast (microscopy)
FITC: fluorescein isothiocyanate
GO: gene ontology
NCBI: National Center for Biotechnology Information
ORF: Open Reding Frame
PCR: polymerase chain reaction
PTw: phosphate buffered saline with 0.1 % Tween-20
PTx: phosphate buffered saline with 0.1 % Triton-X
qPCR: quantitative Real Time PCR
RNA-Seq: RNA sequencing
SD: standard deviation
SEM: scanning electron microscopy
TEM: transmission electron microscopy
