# Supplementary figures and images for: Do novel genes drive morphological novelty? An investigation of the nematosomes in the sea anemone Nematostella vectensis
Source: BMC Evol Biol. 2016 May 23;16:114. doi: 10.1186/s12862-016-0683-3 (PMC4877951; doi:10.1186/s12862-016-0683-3)

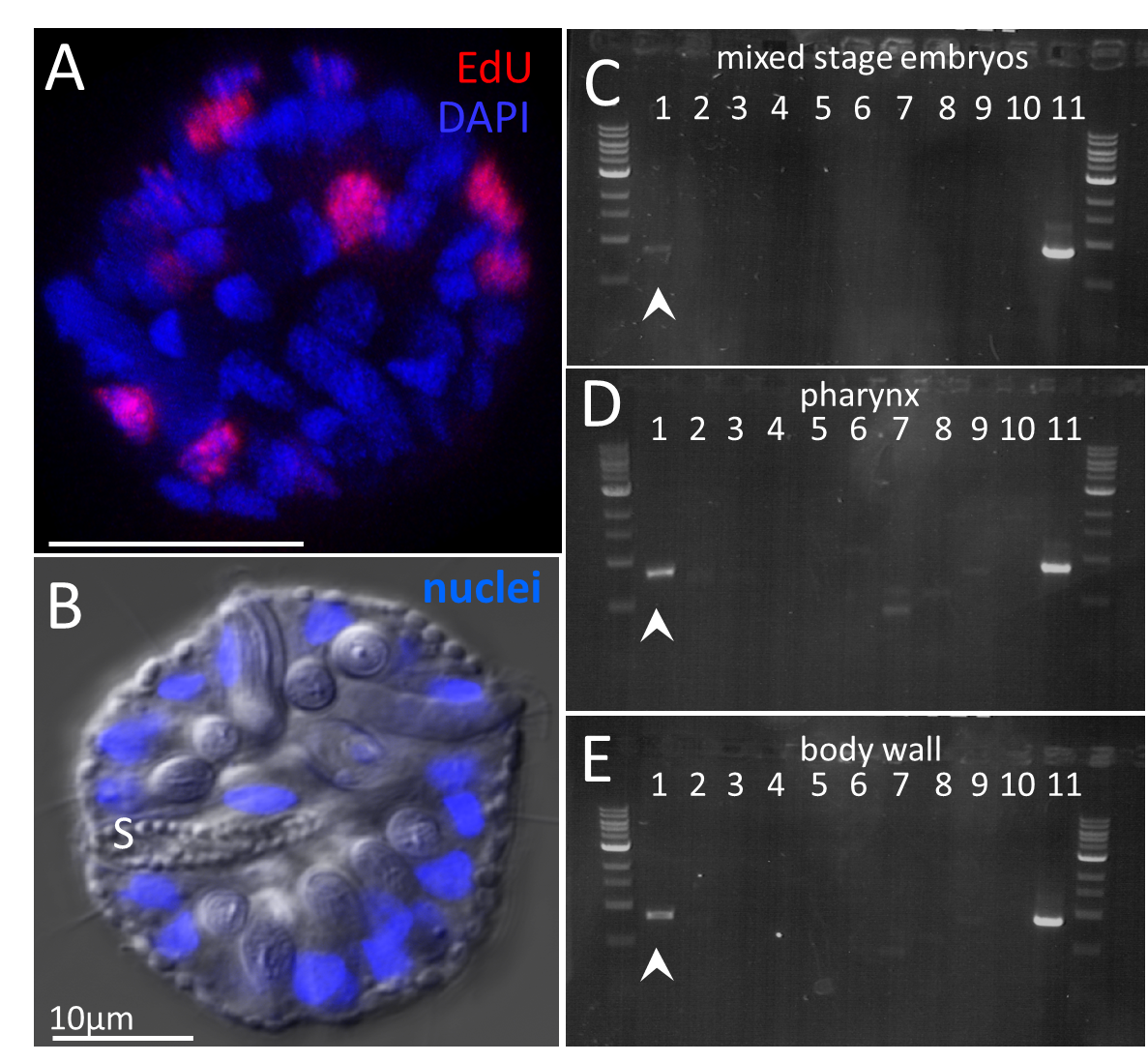

Supplement: Additional file 3: — Further examination of nematosomes. (A) Proliferating cells in an individual nematosome labeled while it was circulating through the body cavity (red - proliferative nuclei, blue - non-proliferative nuclei, 1 μM DAPI). A total of three nematosomes have ever been observed to have proliferating cells via EdU. (B) (C-E) PCR confirms that nematosome-specific transcripts are uniquely expressed in nematosomes. Nine out of ten primers designed to amplify transcripts expressed uniquely in nematosomes failed to amplify products of the predicted size in cDNA samples extracted from mixed stage embryos, adult pharynx, and adult body wall. The product amplified using primers for Nv5804 (lane 1) appears to be ubiquitously expressed across all three tissues assayed. Actin was amplified as a positive control in lane 11. Faint bands in lanes 7-9 of the pharynx are of the wrong size and likely represent mis-priming. Lane 1: Nv5804, 2: Nv4198, 3: Nv13913, 4: Nv18469, 5: Nv5803, 6: Nv5749, 7: Nv9367, 8: Nv19938, 9: Nv18000, 10: Nv2134, 11: actin. See Additional file 8 for primer sequences. (PNG 1597 kb) [file 12862_2016_683_MOESM3_ESM.png]
